# Structures of 1-deoxy-D-xylulose 5-phosphate (DXP) Reductoisomerase (IspC) from *Acinetobacter baumannii* in complex with Potential Inhibitors for Antimicrobial Drug Development

**DOI:** 10.1063/4.0000833

**Published:** 2025-10-27

**Authors:** Meagan Belcher Dufrisne, Misgina Girma, Kyung Hyeon Lee, Soo Hyeon Lee, Iswarduth Soojhawon, Robin Couch, Cynthia Dowd, Schroeder Noble

**Affiliations:** 1 Walter Reed Army Institute of Research, Silver Spring, MD, USA; 2 George Mason University, Department of Chemistry and Biochemistry, Manassas, VA, USA; 3 George Washington University, Department of Chemistry, Washington DC, Washington DC, USA

## Abstract

Drug-resistant bacterial pathogens, including carbapenem-resistant *Acinetobacter baumannii* (CRAB), are a major worldwide health concern. Since *A. baumannii* can survive in biofilms on surfaces for months, civilians and military service members are at higher risk of hospital-acquired CRAB infections during long hospitals stays, or when in need of devices like catheters or ventilators. The dire need for novel therapeutics to treat *A. baumannii* infections is illustrated by the rise of multidrug-resistant (MDR) infections and cases that are completely resistant to available treatments, including drugs considered the last line of defense. Isoprenoids comprise a diverse group of biological molecules, the synthesis of which is essential for basic biological functions. In bacteria, this includes maintenance of the bacterial membrane and cell wall, as well as energy production. The methylerythritol phosphate (MEP) pathway is responsible for the biosynthesis of key isoprenoid precursors and is an essential pathway in bacteria. However, in eukaryotes, isoprenoids are synthesized by an entirely different pathway, making the MEP pathway an exciting target for antibiotic development. IspC is a key enzyme in the MEP pathway that catalyzes the reduction and isomerization of 1-deoxy-D-xylulose 5-phosphate (DXP) to MEP in the second step of the pathway. Fosmidomycin and its analogue FR900098 are known inhibitors of *A. baumannii* IspC (AbIspC) *in vitro*. Our recent work has shown that only FR900098 inhibits growth of the pathogen in CAMHB, but the minimum inhibitory concentration is very high. Based on these results, we sought to further optimize compounds to inhibit AbIspC toward antibiotic development using a structure-based approach. This approach includes iterative lead optimization by medicinal chemistry, structural biology of AbIspC bound to promising compounds and testing of the compounds in enzymatic activity assays. Here, we present structures of AbIspC bound to three different compounds in the presence of Mg^2+^ and cofactor NADPH at 2.06 Å, 1.97 Å, and 2.00 Å resolution. These three compounds (FR900098 derivatives referred to as compound A, B, and C) were found to have IC_50_ values of 154 nM, 47 nM, and 172 nM, respectively. These compounds are promising, and studies to assess the antibacterial activity of these compounds are ongoing. These data provide a springboard for continued structure-based drug design toward our goal of developing a potent IspC inhibitor with improved antibacterial activity against MDR *A. baumannii* strains.

